# Strategic decision making and prediction differences in autism

**DOI:** 10.7717/peerj.13328

**Published:** 2022-04-21

**Authors:** Vasileios Mantas, Artemios Pehlivanidis, Katerina Papanikolaou, Vasileia Kotoula, Charalambos Papageorgiou

**Affiliations:** 1 1st Department of Psychiatry, Eginition Hospital, National and Kapodistrian University of Athens, Athens, Greece; 2 Department of Child Psychiatry, Agia Sophia Children’s Hospital, National and Kapodistrian University of Athens, Athens, Greece; 3 Experimental Therapeutics and Pathophysiology Branch, National Institute of Mental Health, Bethesda, Maryland, USA

**Keywords:** Prisoner’s dilemma, Prediction, Desicion making, Autism

## Abstract

**Background:**

Several theories in autism posit that common aspects of the autism phenotype may be manifestations of an underlying differentiation in predictive abilities. The present study investigates this hypothesis in the context of strategic decision making in autistic participants compared to a control group.

**Method:**

Autistic individuals (43 adults, 35 male) and a comparison group (42 adults, 35 male) of age and gender matched individuals, played a modified version of the prisoner’s dilemma (PD) task where they were asked, if capable, to predict their opponents’ move. The predictive performance of the two groups was assessed.

**Results:**

Overall, participants in the autism group had a significantly lower number of correct predictions. Moreover, autistic participants stated, significantly more frequently than the comparison group, that they were unable to make a prediction. When attempting a prediction however, the success ratio did not differ between the two groups.

**Conclusions:**

These findings indicate that there is a difference in prediction performance between the two groups. Although our task design does not allow us to identify whether this difference is due to difficulty to form a prediction or a reluctance in registering one, these findings could justify a role for prediction in strategic decision making during the PD task.

## Introduction

Autism is a neurodevelopmental condition that is characterised by a diversity of phenotypic features mainly including difficulties in reciprocal communication, social interactions as well as restricted, repetitive patterns of behaviours, interests and activities (American Psychiatric Association, 2013). Within the last decade, a few theories have been proposed, suggesting that some of the perceptual and cognitive differences in autism might be directly or indirectly linked to altered processes of prediction (Pellicano & Burr, 2012; Sinha et al., 2014; Van de Cruys et al., 2014).

The ability to make predictions in our everyday life, is a key characteristic of all adaptive and intelligent systems and allows us to prepare for impeding circumstances (Friston, Kilner & Harrison, 2006). Forming predictions is a complicated process which mostly relies on reward processing and several studies have delineated the many aspects of this process, including reward anticipation, receipt of rewards and reward learning which also includes prediction errors (Olney et al., 2018). Prediction errors occur when there is a difference between received and predicted rewards. They have a powerful influence on reward learning and subsequent behaviour since, if a reward related outcome is more or less valuable than predicted, the association between the stimulus or action and the reward can be modified in order to better learn the former’s predictive value (O’Neill & Schultz, 2018).

As a result, the way prediction errors are processed partly determines the predictive abilities of individuals (Brown & Brüne, 2012). Although key in updating and improving someone’s predictive processes, prediction errors sometimes should be ignored (Mosner et al., 2019). This fine balance between processing and ignoring prediction errors is what undelies the specific autistic characteristics according to the theory of Hightened Precision of Prediction Errors in autism. Specifically, the high and inflexible estimation of precision in prediction errors affects learning and does not allow any flexibility in the environment when predictions are formed (impaired predictions) (Van de Cruys et al., 2014).

Alternatively, Pellicano & Burr (2012) conceptualised specific autistic features by assuming broader and weaker predictions that lead autistic individuals to perceive the world more accurately and their perceptions to be less influenced by previous experiences. This concept has laid the foundation for the Prediction Impairment in Autism (PIA) hypothesis. The PIA hypothesis states that autism is characterised by a reduction in one’s sensitivity to relationships that are weak and/or are characterised by longer temporal spans (Sinha et al., 2014). As a result, some inter-event relationships which might appear evident to non autistic individuals are invisible to those with autism, leading to impaired predictions (for experimental evidence around the PIA hypothesis, see the review by Cannon et al., 2021).

These theories make prediction differences, key for understanding autism and could explain the social, cognitive as well as physical autism characteristics including motor, language difficulties, social interactions and theory of mind deficits including deficits in decision making.

Decision making is a complex mental process and autistic individuals report experiencing difficulties when taking decisions more often than those without the condition (Johnson et al., 2006; Luke et al., 2012). Studies utilising standard, non-social decision making tasks such as the Iowa gambling task have indeed evidenced atypical responses in autism where participants tend to be slower in taking a decision and demonstrate a more logical approach and care in making a choice (Vella et al., 2018). Additionally, when asked to judge the accuracy of their decision, autistic participants often differ in their levels of confidence compared to the control group, despite their decisions being actually correct (Doenyas et al., 2019; Sahuquillo-Leal et al., 2020). These unique autism characteristics of the decision making process are more prominent in social settings and when moral subjects are concerned. In the present study, we have utilized the Prisoner’s Dilemma (PD) task in order to examine whether prediction difficulties in autism would influence strategic decision making as this is employed by the task.

The PD task is a well-known model in game theory, where strategic interactions of individuals are modeled in situations containing specific rules and outcomes. In the PD task, the player needs to decide whether to cooperate with their opponent or defect and gain the maximum payoff. The task represents the conflict between the optimum distribution of rewards (Pareto optimal) and the optimum choice for an individual (Nash equilibrium) (Apt & Grädel, 2011) It is considered an analogue of social interaction and as such it has been used a lot in autism research where social behaviour and interactions are key characteristics of the disorder. Findings concerning the PD task in autism, indicate that, cooperation could improves with age (Jin et al., 2020; Kaartinen et al., 2019) and the development of executive functions (Li, Zhu & Gummerum, 2014) and that reputation (Maurer et al., 2018) and the nature of the opponent (actual human or machine) could also influence cooperation. These factors are important for both autistic (Sally & Hill, 2006) and non autistic individuals (Mienaltowski & Wichman, 2020; Milinski & Wedekind, 1998; Wang et al., 2012).

As far as decision making is concerned, the task involves making a decision about whether to defect or cooperate, anticipate the opponent’s decision and getting the final feedback for the player’s choice, in the form of points (Thompson et al., 2021). The ability to predict the opponent’s move could thus influence task performance. Given the theoritical frameworks that place prediction differences in autism as a central component that could underlie a lot of the characteristic behaviours in autism including differences in decision making, we aim to investigate prediction differences in strategic decision making with the use of the PD task.

For that purpose, we have modified the PD task and included a prediction component where participants after deciding on their task move, are asked whether they could register a prediction concerning their opponent’s move. We hypothesise that autistic individuals would make fewer correct predictions concerning their opponent’s move. Additionally, as a result of their prediction differences we expect participants in the autism group to show altered performance in the PD task.

## Materials and Methods

### Participants

This study recruited autistic adults with no intellectual disability (43 adults, 35 male) and age-matched non autistic volunteers (42 adults, 35 male). For details see Table 1. The autistic participants were recruited from a larger cohort of volunteers (Pehlivanidis et al., 2020) who participated in a research project on the *de novo* diagnosis of adults with neurodevelopmental disorders. With the term *de novo* we refer to adult participants who were diagnosed for the first time in their lives as part of the aforementioned project (Pehlivanidis et al., 2020). Recruitment for our study took place at the Adult Neurodevelopmental Outpatient Clinic of the 1^st^ Department of Psychiatry of the National and Kapodistrian University of Athens. Patients who visit the clinic are either self referrals who were willing to receive an autism diagnosis or referrals from other health services. Autism diagnosis was based on DSM-5 criteria and all autistic participants had fluent phrase speech and more than 12 years of education. Exclusion criteria included the presence of acute psychopathology, systematic psychopharmacological treatment up to 30 days prior to taking part in the study as well as current substance abuse disorder as assessed by the medical history of the participants. Finally, IQ < 70 assessed with Wechsler Adult Intelligence Scale (WAIS-IV) (Wechsler, 2008), any known genetic condition and past knowledge of the PD task were exclusion criteria for study participation.

**Table 1:** Group characteristics. The characteristics of our autism and control groups are presented in this table. IQ scores, as measured by the WAIS-IV are significantly diﬀerent between the two groups and as a result they were included as a covariate in all our analyses.

	Autism	Control	*p*-value*
**Age mean** (std)	28 (9)	26 (5.8)	>0.5
**Gender *n*** (%)			
Male	35 (81)	35 (83)	
Female	8 (19)	7 (17)	
**IQ mean** (std)	105 (13.4)	113 (11.6)	<0.5

**Note:**

* Mann–Whitney U was used when non-normality could not be excluded, else ANOVA.

The comparison group was recruited *via* advertisement at the National and Kapodistrian University of Athens by word of mouth and included individuals without neurodevelopmental disorders with IQ > 70 (WAIS-IV) and more than 12 years of education. Exclusion criteria also included the presence of acute psychopathology as assessed by the medical history of participants, and past knowledge of the PD task. Written informed consent was obtained from all participants and the study was approved by the Ethics Committee of the Department of Psychiatry, National and Kapodistrian University of Athens (IRB 12/7/2018 #517).

### The PD task

Our own computerized version of an iterated, simultaneous PD task was developed for this study (Mantas et al., 2022a). A simultaneous PD involves the participant and their opponents submitting their task choices at the same time, without knowing what each other chose to do (for review see Mantas et al., 2022b). Each participant played against four virtual opponents. During the PD task, participants are asked to choose between cooperation and defection while unaware of their opponent’s move. If both participants cooperate, they each get three points (R-Reward). If only one cooperates, then this player gets zero points (S-Sucker’s payoff) while the defector gets five (T-Temptation). If they both defect, they get one point (P-Punishment) each. In PD the payoffs satisfy T > R > P > S and different strategies can be implemented to examine different aspects of behavior during the task (Bravetti & Padilla, 2018).

In our version of the PD task, the opponents’ order was randomized for each participant and each opponent had a different strategy: Tit-for-Tat (TFT) (Axelrod & Hamilton, 1981), Win-Stay-Lose-Shift (WSLS) (Nowak & Sigmund, 1992), Always-Cooperate (AC) and Always-Defect (AD). TFT is a strategy in which the participant mimics the action of their opponent after cooperating in the first round while WSLS involves choosing the same response as long as these are rewarded and switching to a different task choice once this is no longer the case. Different PD strategies were included in order to create a representative analog of the pluralistic, social, real-life environment (for more information on the PD strategies please see the review by Mantas et al., 2022b). The number of rounds was set to twenty for the TFT and WSLS strategies. For AC and AD strategies the number of rounds was set to ten as they are simpler, and the opponent’s response is always the same. The players were not informed about the exact round number of each game but were notified when the last round began. Opponents were represented as avatars with random appearance and sex and participants were informed that the opponent’s behavior simulated real players’ strategies.

Participants were instructed to achieve the highest possible score (total sum of gained points). Available choices, ‘cooperate’ and ‘betray’, were symbolized by ‘paper’ and ‘scissors’, respectively. These symbols were chosen since they lack the social bias of the words ‘cooperation’ and ‘betrayal’, they are simple and easily conceptualised as part of a famous game (rock-scissors-paper).

For the purpose of this experiment which focused on the prediction abilities of the autism group compared to the control group, during strategic decision making, the PD task was modified by adding a prediction component. At each round of this task and after deciding whether they would defect or cooperate, participants were asked to register whether they could predict or not their opponent’s move. If they registered that they were able to make a prediction, they had to submit their exact prediction. No time frame was imposed within which participants had to submit their prediction (see Supplemental Material).

The task procedure was fully explained, and participants were encouraged to ask questions. Subsequently, each participant played two example games with random opponent decisions in order to familiarize themselves with the task. At the end of the example games, players were asked task-relevant questions in order to ensure understanding of the task requirements. These questions included when participants had to make their task choice, what the ‘paper’ and ‘scissors’ symbols were standing for and what each choice represented in terms of rewards as well as the fact that they would be asked to predict their opponent’s move. If they answered wrongly, the task was explained again and the procedure was repeated. Task exclusion criteria included failure to understand the task, no prediction attempts or failure to make any correct predictions. No participants were excluded from this study.

The experiment took place at a specially assigned research room at the Adult Neurodevelopmental Outpatient Clinic of the 1^st^ Department of Psychiatry of the National and Kapodistrian University of Athens. To ensure data quality and consistency, all the data were acquired by the same experimenter who was present in the room but apart from explaining the task and ensuring sufficient understanding of the task procedure, had minimal interaction with the participants. Participants registered their task responses using a ten-button key board where the two keys used for task responses were marked with the scissors and paper symbols. The same key board was used in order to register their predictions concerning their opponent’s move.

### Analysis

Task performance, as indexed by the cooperation levels and total number of points won was calculated for the autism group and the control group. The number of rounds where participants registered that they were confident to and attempted a prediction, the ratio of wrong over attempted predictions and the number of correct predictions were used to assess the predictive abilities of our groups. Since the total number of rounds is kept fixed for all participants for both groups, and prediction difficulties would be indexed as incorrect predictions or the number of rounds where an inability to predict was declared, we use the number of correct predictions, to asses any difficulties in the predictive abilities between the autistic participants and the control group. The total number of correct predictions is what remains when the rounds where participants declared an inability to make a prediction and incorrect predictions are subtracted from the total number of rounds.

Simple linear regression was used to compare task performance and the prediction abilities of the autism group and the comparison group. Statistical analysis of the task data was performed using in house code written in Python (v3.8.11) and R (v4.0.2). Since the two groups were significantly different in their IQ scores (see Table 1), we examined the role of diagnosis in the prediction performance of the two groups, including the IQ score as a covariate. Scales such as the WAIS, usually underestimate the IQ scores of autistic individuals (O Saad, L., 2018; Soulières et al., 2011) and in the literature it has been debated whether or not IQ scores should be included as covariates in autism studies (Dennis et al., 2009). In our analyses however, we decided to include IQ as a covariate and we would like to acknowledge that this could perhaps obscure the role of autism in our results.

## Results

In order to examine the prediction abilities of the autism group, the total number of correct predictions between autistic participants and the control group (see Table 2) was examined using simple linear regression (*F*(2,82) = 20.03, *p* < 0.001) and diagnosis was a significant predictor (*β* = −6.59, *p* = 0.009, SE = 2.49) accounting for a mean of approximately seven fewer correct predictions in the autism group than the comparison group. This finding indicates that overall, the autistic participants made fewer correct predictions for their opponent’s move.

**Table 2:** Group task performance. This table provides a summary of the PD performance for our two groups. These metrics were used in a linear regression model in order to examine the role of the ASD diagnosis in the PD task.

	Autism	Control
**Correct predictions**		
Mean	27.9	37.9
Median(iqr)	30(12.5–41)	39.5(31.2–44.75)
**Rounds declared inability to predict**		
Mean	21.1	10.6
Median(iqr)	15(7–31)	9.5(6–15.25)
**Failed over total attempted predictions**		
Mean	0.28	0.2
Median(iqr)	0.23(0.14–0.42)	0.19(0.12–0.27)

To further examine this finding simple linear regression was used (*F*(2,82) = 16.4, *p* < 0.001) to compare the total number of rounds where participants declared incapability to make a prediction (see Table 2). Diagnosis was a significant predictor (*β* = −7.26, *p* = 0.009, SE = 2.72) and autistic participants were unable to make a prediction in approximately seven more rounds compared to non-autistic participants. When the ratio of wrong attempted predictions over the total number of attempted predictions was compared between the two groups, using simple linear regression (*F*(2,82) = 6.172, *p* = 0.003), it did not reveal any significant difference (*β* = 0.05, *p* = 0.088).

No significant differences were identified in the total score (simple linear regression, *F*(2, 82) = 2.914, *p* = 0.059) and cooperation levels (simple linear regression, *F*(2, 82) = 0.4798, *p* = 0.62), between autistic individuals and the comparison group (see Table 2). Our sample size, however, does not allow us to firmly conclude on any absence of difference in task performance. Finally, as part of an exploratory analysis, we present the total number of correct predictions that each group made for each of the four PD strategies (see Table 3).

**Table 3:** The table presents the results per opponent’s strategy. The analysis followed was identical to our main analysis as described in the manuscript. The β-coef corresponds to the contribution of ASD diagnosis as a feature of the analysis. The code and dataset used for this analysis is included in the supplementary material.

Opponent’s strategy	Factor	β-coef	SE	*p*-value
Tit-for-tat	Correct predictions	−2.09	1.04	<0.05
	Rounds declared inability to predict	1.87	0.97	>0.05
	Failed over total attempted predictions	0.05	0.04	>0.05
	Total score	0.38	2.56	>0.05
	Cooperation level	−0.14	1.36	>0.05
Win-Stay-Lose-Shift	Correct predictions	−2.12	1.13	>0.05
	Rounds declared inability to predict	2.07	1.10	>0.05
	Failed over total attempted predictions	0.01	0.05	>0.05
	Total score	−1.42	1.46	>0.05
	Cooperation level	0.39	1.21	>0.05
Always-Cooperate	Correct predictions	−1.75	0.53	<0.01
	Rounds declared inability to predict	1.60	0.59	<0.01
	Failed over total attempted predictions	0.12	0.05	<0.05
	Total score	−1.86	1.44	>0.05
	Cooperation level	0.93	0.72	>0.05
Always-Defect	Correct predictions	−1.71	0.53	<0.01
	Rounds declared inability to predict	1.23	0.51	<0.05
	Failed over total attempted predictions	0.09	0.05	>0.05
	Total score	0.25	0.39	>0.05
	Cooperation level	−0.25	0.39	>0.05

## Discussion

The present study investigates the prediction abilities of autistic individuals compared to non-autistic individuals using a strategic decision making task, namely the Prisoner’s Dilemma. For this purpose, autistic participants with no intellectual disability and age and gender matched non-autisic volunteers played a modified version of the PD task. At each round of this task and after deciding whether they would defect or cooperate, participants had to register, without any time restrictions, whether they could predict or not their opponent’s move.

The total number of rounds, where participants registered that they were unable to make a prediction as well as the total number of correct predictions were examined separately and compared between autistic individuals and the control group. It was shown that participants in the autistic group registered an inability to make a prediction for their opponents’ move in statistically significantly more rounds. Additionally, when the number of correct predictions was compared between the two groups, autistic participants had a statistically significantly lower number of correct responses. These findings, at first glance, appear in line with our hypotheses and the prediction theories in autism since the hypothesised differential predictive abilities between the two groups, were expressed as altered performance in prediction during decision making. When a prediction was attempted, however, the success rate did not differ between autistic participants and the control group.

The design of our PD task, we believe, allows differences in prediction performance to be expressed as 1. reduced prediction accuracy, demonstrated as registered but wrong predictions and 2. a difficulty or reluctance to form a prediction which could be expressed in our task by answering negatively to the question: “Can you predict your opponent’s move?”. Reluctance and caution in decision making have previously been reported in the autism literature. Autistic individuals are more strongly motivated by their fear of failure than sensitivity to reward (South et al., 2011). Indecisiveness in young adults with Asperger Syndrome is also reported by parents (Johnson et al., 2006). Additionally, autistic participants employ a more deliberate and rule-based strategy with longer decision-making times and longer event-related potential latencies (McPartland et al., 2004), in contrast to a more intuitive strategy that uses a jumping to conclusions pattern (Brosnan, Chapman & Ashwin, 2014).

Reluctance in decision making could be attributed to the lower confidence levels that usually characterise autistic individuals and especially children (Doenyas et al., 2019; Grainger, Williams & Lind, 2016; Sahuquillo-Leal et al., 2020; Sawyer, Williamson & Young, 2014; Wilkinson et al., 2010; Wojcik et al., 2011). These studies have utilised decision making tasks and have shown that when autistic participants were asked to assess their level of confidence concerning their task choices, their assessment was significantly different to that of the controls. Their confidence levels, however, did not predict task performance (Doenyas et al., 2019; Grainger, Williams & Lind, 2016; Sahuquillo-Leal et al., 2020). Our results would benefit from a comparison of task performance between our groups, as this would allow us to further clarify whether prediction differences are due to reluctance or not. We do not however, have enough power to draw conclusion from such an analysis.

**Figure 1:**
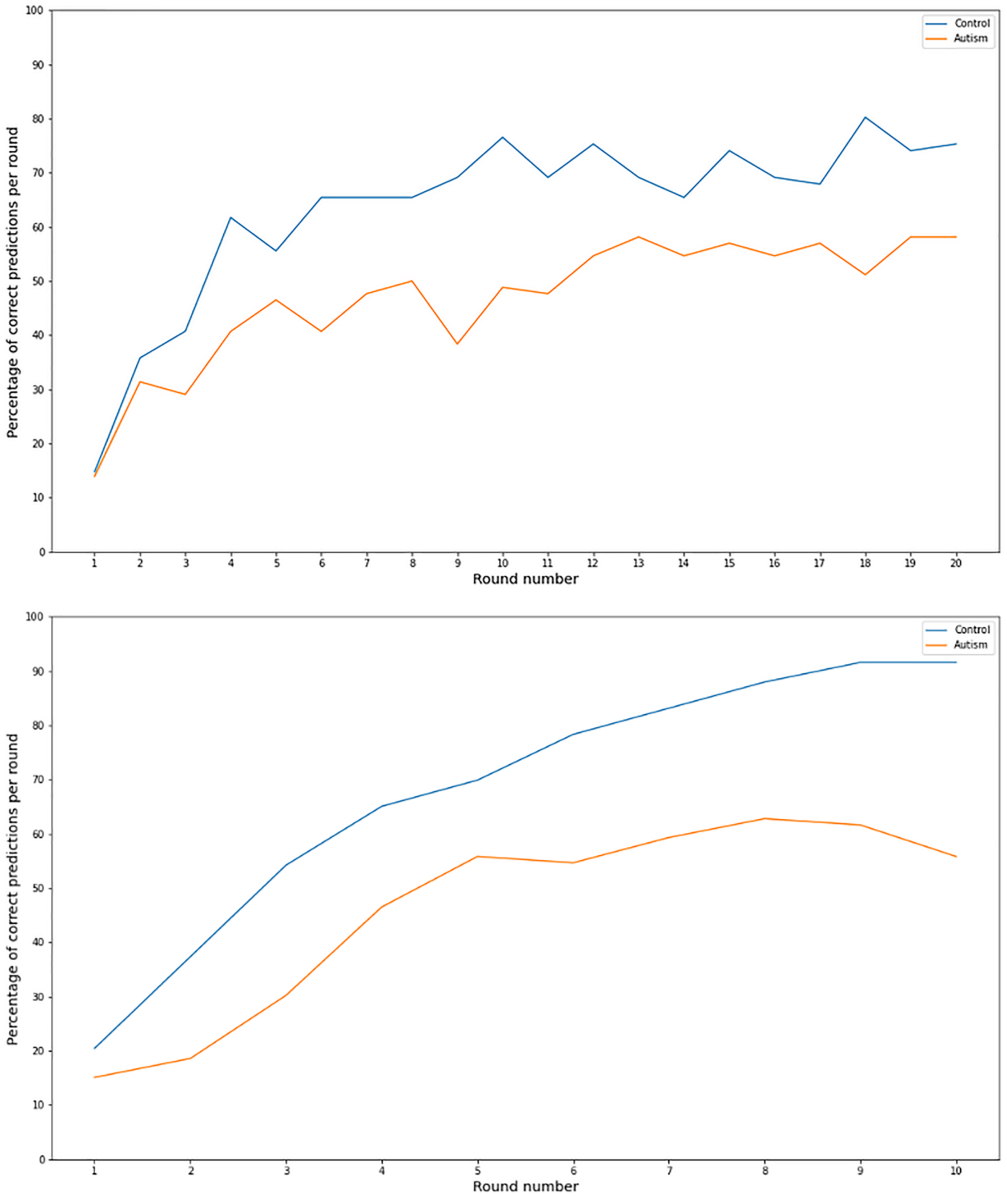
Correct prediction per task round for 10 and 20 round games. Line graph of the percentage of correct predictions per round number for the 20 (upper graph) and 10 (lower graph) rounds games for the autism and the comparison group.

According to experimental evidence (Brosnan, Chapman & Ashwin, 2014; Schuwerk, Sodian & Paulus, 2016), a more cautious decision making in autism could not only be the result of low confidence but also lack of sufficient information. In line with this, the differences in the prediction accuracy between autistic and non-autistic participants, observed in our study, could be attributed to the fact that the autism group did not have sufficient information to predict their opponent’s move. In such a scenario, however, as the number of task rounds increases, the per round predictive performance of autistic participants would also get closer and finally reach that of the control group. A similar pattern is not observed in our data (Fig. 1). Moreover, in our PD design, no time constraint was imposed to participants when asked to register their predictions for their opponent’s move and task choices, excluding time limitations as a factor that would contribute to reluctance and thus altered predictions in our version of the task.

If on the other hand, a total prediction impairment was present in our autism group, this would lead to the group employing alternative ways in order to reach a PD decision. These methods could include making random choices or other unorthodox ways of deciding. In our task, in the autism group and the comparison group, predictive performance improves (Fig. 1) with time, making it very unlikely that their responses are due to chance.

To summarise, our data do not allow us to disentangle whether the diminished predictive performance of our autism group is due to an actual difficulty in their ability to form predictions or due to cautiousness and reluctance in registering an already formed prediction. Such a distinction would be possible in our study, only if participants were given no alternative but to register a response concerning the opponents move. In that scenario, fewer correct predictions would allow us to conclude that the differences in the prediction performance of autistic individuals is indeed due to prediction inabilities and not due cautiousness.

Our study has several limitations. Our sample consists of individuals with high functioning fluent who were diagnosed for the first time in their lives as part of a larger project (Pehlivanidis et al., 2020). Despite its homogeneity, this unique characteristic of our sample limits the generalisability of our findings that might not apply to autism groups with more severe and/or diverse characteristics. In our study, we did not have a big enough sample to determine whether there was a difference in task performance between autistic participants and the control group. Potential differences in task performance, however, could be important in interpreting the differential predictive abilities of our groups. As a result, our findings need to be further supported by future studies with bigger samples. Additionally, and although our data suggest otherwise, we cannot exclude the possibility that the number of rounds of the PD experiments could limit our ability to decipher whether the diminished prediction performance of our sample could be overcome by adding more rounds to each PD game. Finally, our task performance could be altered if participants, were rewarded or punished for their choices concerning their predictions. On one hand, this could have improved task performance as it would have provided additional motivation. On the other hand, it might have lead to the autistic participants harnessing alternative mechanisms to overcome and disguise their prediction abilities which was the point of focus of our study.

## Conclusions

To our knowledge, this is the first study testing the role of prediction in strategic decision making of autistic individuals. For that purpose, we have used a modified version of the PD task where participants are asked to register a prediction concerning their opponents’ move. Our findings indicate differential prediction abilities for autistic participants who engage in strategic decision making. Our version of the PD task, however, does not allow us to examine which aspects of the prediction mechanism are responsible. This could be possible by conducting more future studies that would help characterise in more detail the prediction differences observed in strategic decision making in autism.

## Supplemental Information

10.7717/peerj.13328/supp-1 Supplemental Information 1 Study dataset.

10.7717/peerj.13328/supp-2 Supplemental Information 2 Code for analysis. Jupiter notebook files with the code for data analysis

10.7717/peerj.13328/supp-3 Supplemental Information 3 Written task instructions for participants (translated).

10.7717/peerj.13328/supp-4 Supplemental Information 4 Written task instructions for participants in Greek.

10.7717/peerj.13328/supp-5 Supplemental Information 5 PD task screenshots. Screenshots of the PD task as it was presented to participants

## Additional Information and Declarations

### Data Availability

The following information was supplied regarding data availability: The anonymized dataset with participants’ groups characteristics and task relevant information used in analysis are available in the Supplemental File.
