# Supplementary material for: Strategic decision making and prediction differences in autism
Source: PeerJ. 2022 Apr 21;10:e13328. doi: 10.7717/peerj.13328 (PMC9035278; doi:10.7717/peerj.13328)
Supplement: Supplemental Information 3 [file peerj-10-13328-s003.doc]

**Instructions for the Prisoner’s Dilemma Task**

Throughout life, we make decisions that affect and are affected by, others.

The level οf success of someone’s goals in society, is at least partially considered the result of these decisions.


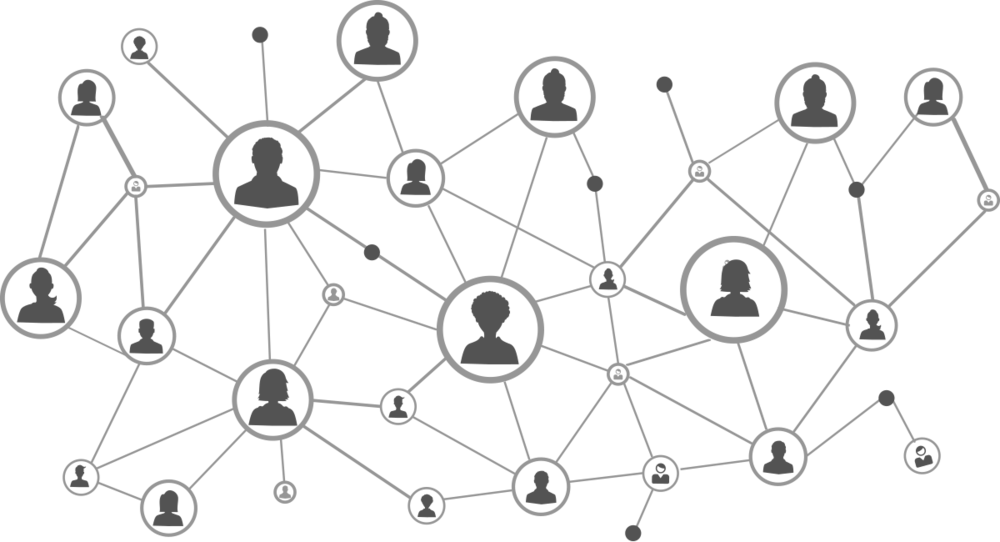


Our decisions, reflect our character and create new perspectives. They reflect our personal strategy which is differentiated depending on our goal, our ‘opponent’ and the circumstances. Therefore, each choice is the result of personality traits, the history of people’s previous decisions, how much we trust them, the estimated future consequences, the long or short term perspective of our goals and more.

As a result of multiple components effect, available choices in an occurring dilemma differentiate according to circumstances, while often, paradoxical dynamics arise with regard to each party’s choices. This concept is easily understood in the socioeconomic arena.

In a typical example, two competitive corporations have to decide, without knowing the intentions of one another, whether or not to advertise their products for the season. Advertisement is expensive but also very promising for profit.

If both decide to advertise, they will bear the cost of the advertisement without the benefit from it, as there will be neutralization of advantage from each others advertisement. The profit will be equally shared but reduced.


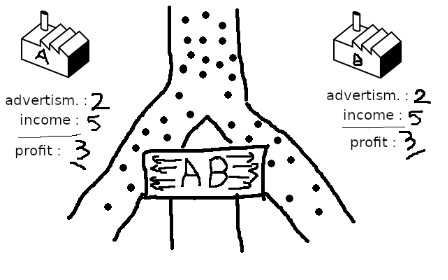


If only one of the two corporations advertises, it will gain the most profit against its competitor.


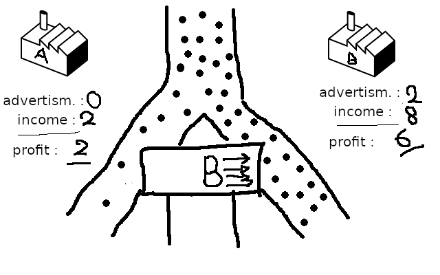


Finally, if both choose not to advertise, nobody will have to bear the cost of it while they will share the profit.


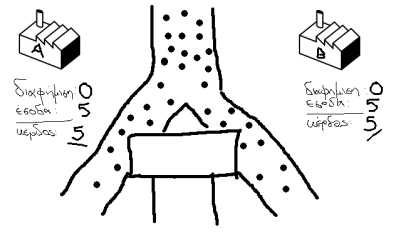


You will play a game, through a computer. You will confront various virtual artificial intelligent opponents, one at a time, for a different number of rounds. You cannot know the number of rounds but you will be informed about the last round of each game. In each round, you and the opponent, have to pick one of the following, ‘scissors’ or ‘paper’.


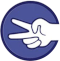

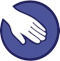


After making a decision, the choices made are revealed simultaneously to both players. Depending on the decisions’ combination there will be a reward for each party.


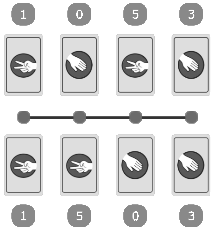


If you both choose ‘scissors’ you win 1 point each, if you both choose ‘paper’ you win 3 points each. Otherwise, whoever chooses ‘scissors’ wins 5 points while the other has no reward.

Each opponent implements his own strategy, unknown to you. Each strategy is a simulation of real human strategy, therefore you are encouraged to interpret opponents as real persons. Depending on the strategy, to calculate their next move, the opponent may take into account your previous decisions. For better performance it is recommended that you keep in mind the previous rounds too.

Moreover, during rounds, you will have to answer whether or not you are able to predict the opponent’s next move and what you think his choice will be.


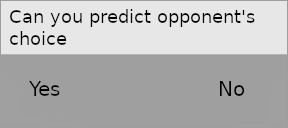

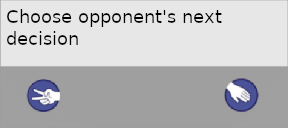


Parties scores, yours and your opponent’s, are presented on the game screen. Your score is the sum of points you gathered from all opponents you have already played with. The game ends when you have confronted all the opponents. Its duration is about 15 minutes. The goal is to gather most points.


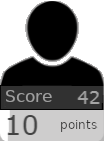


Prior to starting there will be trial rounds for you to get used to the environment and the rules of the game. You will then have the opportunity to pose any question you have.
