# Supplementary material for: Strategic decision making and prediction differences in autism
Source: PeerJ. 2022 Apr 21;10:e13328. doi: 10.7717/peerj.13328 (PMC9035278; doi:10.7717/peerj.13328)
Supplement: Supplemental Information 4 [file peerj-10-13328-s004.doc]

Συνεχώς, στη ζωή μας λαμβάνουμε αποφάσεις οι οποίες, όπως και τα αποτελέσματά τους, επηρεάζουν και επηρεάζονται από τρίτους. Ο βαθμός στον οποίο κάποιος επιτυγχάνει τους στόχους του μέσα στο κοινωνικό σύνολο θεωρείται πως είναι εν μέρει αποτέλεσμα αυτών των αποφάσεων.


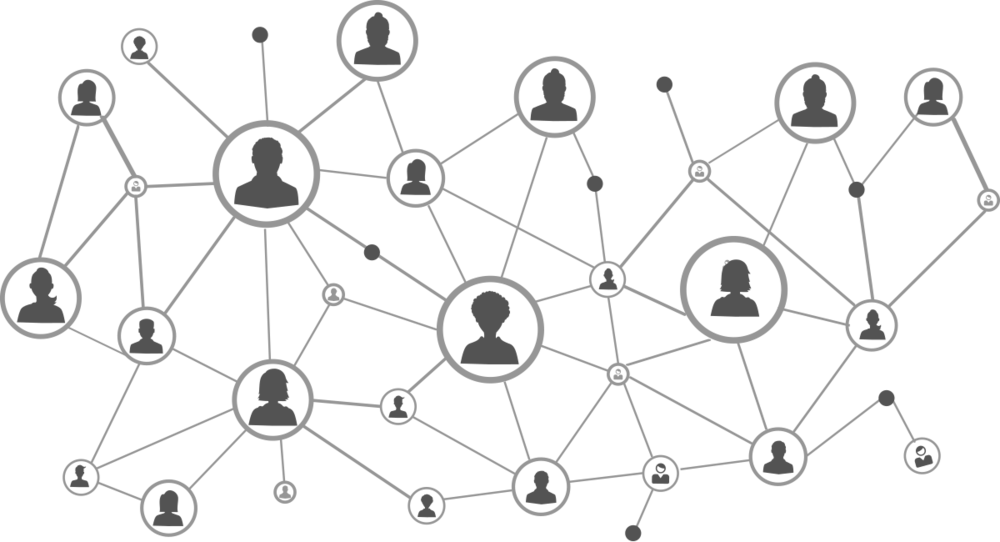


Οι αποφάσεις μας, αντικατοπτρίζουν τον χαρακτήρα μας και δημιουργούν νέες προοπτικές. Είναι εκφραστές της προσωπικής κάθε φορά στρατηγικής η οποία διαφοροποιείται ανάλογα με το στόχο, τον ‘αντίπαλο’ και τις συνθήκες. Έτσι η επιλογή κάποιου είναι συνισταμένη χαρακτηριστικών της προσωπικότητάς του, του ιστορικού των επιλογών των άλλων, του βαθμού εμπιστοσύνης σε αυτούς, των εκτιμώμενων μελλοντικών επιπτώσεων, του μακρόπνοου ή πρόσκαιρου χαρακτήρα του στόχου κ.α.

Αποτέλεσμα των πολλαπλών συνιστωσών, οι επιλογές του καθενός στα προκύπτοντα διλήμματα διαφοροποιούνται ανά περίπτωση ενώ πολύ συχνά δημιουργούνται παράδοξα δυναμικά σε επίπεδο εκατέρωθεν αποφάσεων τα οποία είναι αμφότερα επιζήμια. Αυτό γίνεται εύκολα αντιληπτό στον κοινωνικοοικονομικό στίβο.

Σε ένα τυπικό παράδειγμα, δύο ανταγωνίστριες επιχειρήσεις καλούνται να αποφασίσουν, χωρίς να έχουν γνώση των προθέσεων η μία της άλλης, αν θα διαφημίσουν το προϊόν τους την ακόλουθη περίοδο. Η διαφήμιση κοστίζει μεν, είναι εν δυνάμει προσοδοφόρος δε.


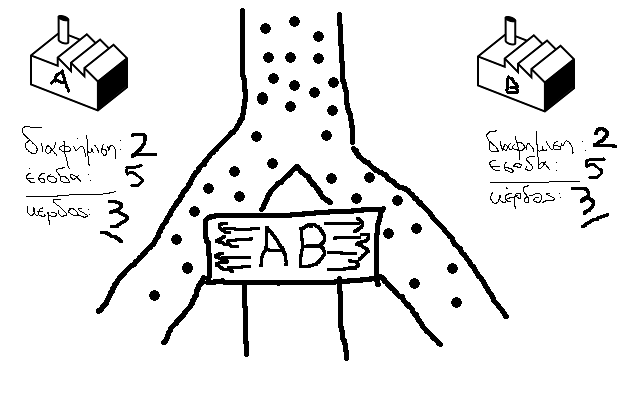


Αν αποφασίσουν και οι δύο να διαφημιστούν, θα υποφέρουν το κόστος της διαφήμισης χωρίς το όφελος αυτής αφού θα υπάρχει αλληλεξουδετέρωση του τελευταίου με τα κέρδη να είναι μοιρασμένα.


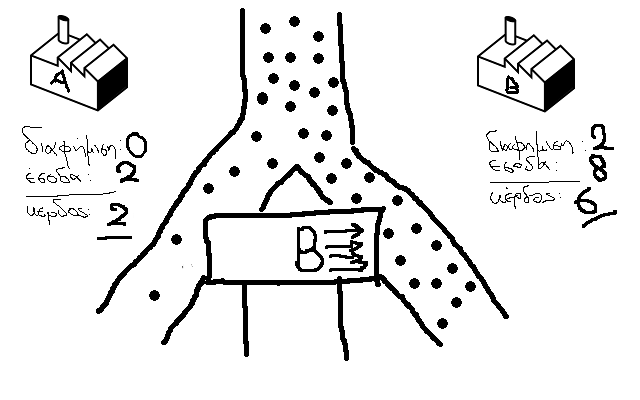


Αν μόνο η μία διαφημιστεί, θα αποκομίσει κέρδος εις βάρος της ανταγωνίστριας.


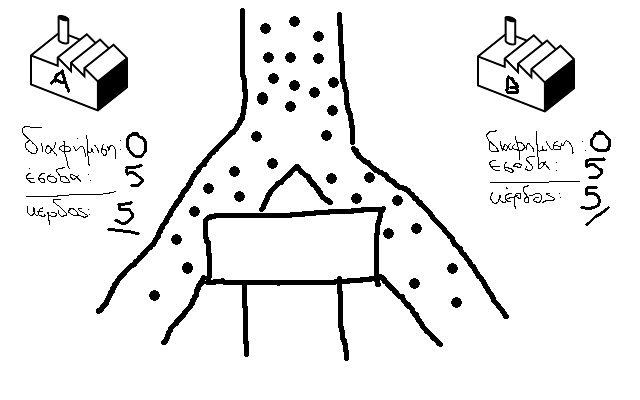


Τέλος, αν και οι δύο δεν επιλέξουν διαφήμιση, δε θα επιβαρυνθούν με το κόστος αυτής ενώ θα μοιραστούν ουσιαστικά και τα κέρδη.

Θα σας χορηγηθεί μία δοκιμασία, ένα παιχνίδι, μέσω ηλεκτρονικού υπολογιστή. Θα αντιμετωπίσετε διάφορους αντιπάλους για ένα διαφορετικό κάθε φορά αριθμό γύρων. Σε κάθε γύρο, καλείστε να επιλέξετε ανάμεσα σε δύο σχήματα, ‘ψαλίδι’ και ‘χαρτί’, το ίδιο και ο αντίπαλος.


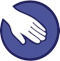

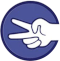


Οι επιλογές αυτές εμφανίζονται και γνωστοποιούνται σε αμφότερους ταυτόχρονα, μετά την παγίωσή τους. Ανάλογα με το συνδυασμό των επιλογών υπάρχει κάποιο κέρδος για κάθε έναν από τους παίχτες.


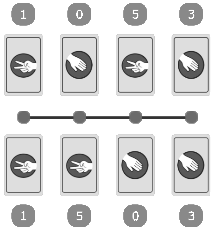


Αν και οι δύο διαλέξετε ‘ψαλίδι’ κερδίζεται από 1 βαθμό, αν και οι δύο διαλέξετε ‘χαρτί’ κερδίζεται από 3 βαθμούς, διαφορετικά όποιος διαλέξει ‘ψαλίδι’ κερδίζει 5 βαθμούς σε αντίθεση με τον αντίπαλο που δεν έχει κέρδος.

Κάθε αντίπαλος, ακολουθεί τη δική του στρατηγική, άγνωστη σε εσάς. Ανάλογα με τη λογική της στρατηγικής, για τον υπολογισμό της επόμενης κίνησης του αντιπάλου, πιθανώς λαμβάνονται υπόψιν και προηγούμενες δικές σας απαντήσεις. Για το βέλτιστο αποτέλεσμα από μέρους σας, συστήνεται ο συνυπολογισμός προηγούμενων γύρων στην τελική απόφαση.

Επιπλέον κατά τη διάρκεια των γύρων, ενδεχομένως να κλιθείτε να απαντήσετε στο αν μπορείτε να προβλέψετε την επόμενη απάντηση του αντιπάλου σας και ποια είναι αυτή.


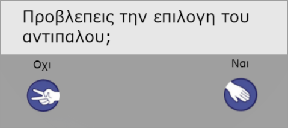

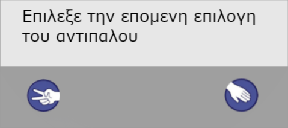


Η βαθμολογία του κάθε παίκτη απεικονίζεται στην οθόνη, το ίδιο και η συνολική βαθμολογία σας, η οποία αποτελεί το άθροισμα των επιμέρους βαθμολογιών που έχετε επιτύχει με τους αντιπάλους.

Η δοκιμασία ολοκληρώνεται όταν αντιμετωπίσετε όλους τους αντιπάλους. Συνήθως, διαρκεί μισή ώρα περίπου.


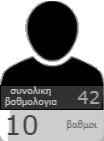


Πριν την έναρξη της δοκιμασίας θα υπάρξουν δοκιμαστικοί γύροι εξοικείωσης υπό την επίβλεψη του υπευθύνου και θα δοθούν διευκρινίσεις όπου χρειαστεί.
