# Supplementary material for: Strategic decision making and prediction differences in autism
Source: PeerJ. 2022 Apr 21;10:e13328. doi: 10.7717/peerj.13328 (PMC9035278; doi:10.7717/peerj.13328)
Supplement: Supplemental Information 5 — Screenshots of the PD task as it was presented to participants [file peerj-10-13328-s005.docx]

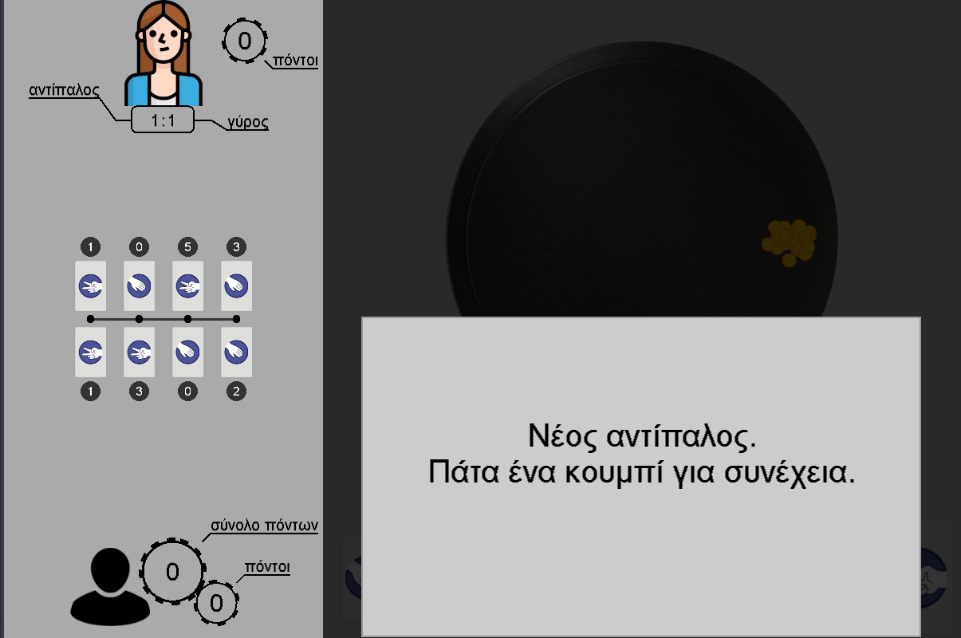


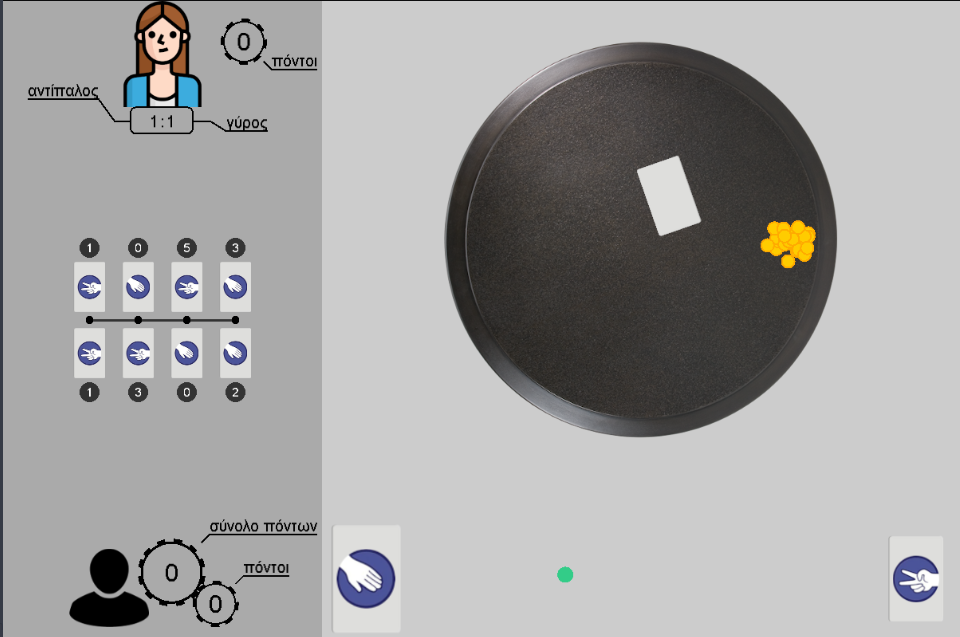


The opponent’s avatar appears on the top left part of the screen and the participant’s at the bottom left part. The round number and the total number of points are shown for the two players. Participants are notified that there is a new opponent and they are asked to press a button to continue (top figure panel).


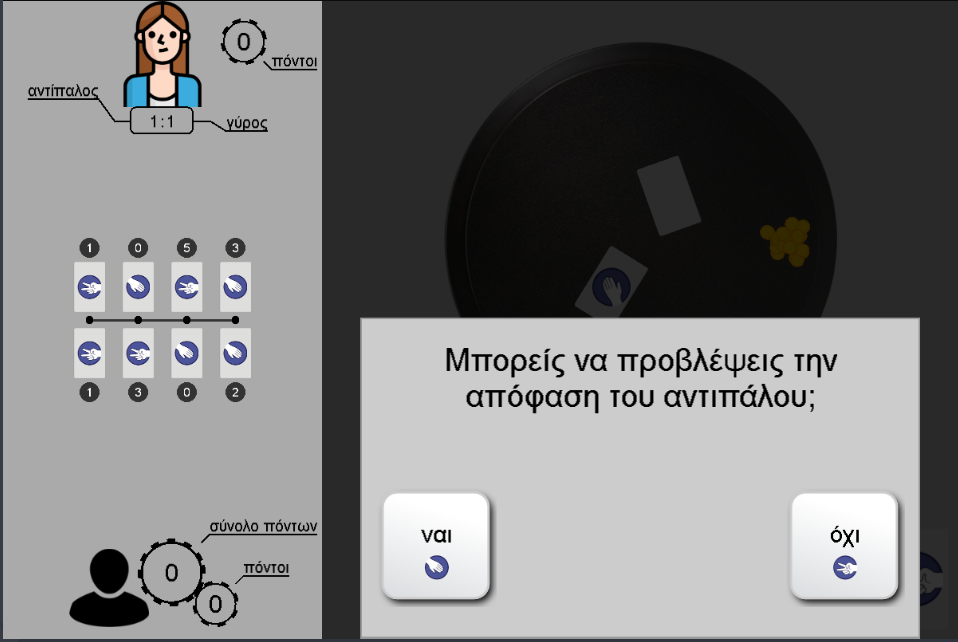


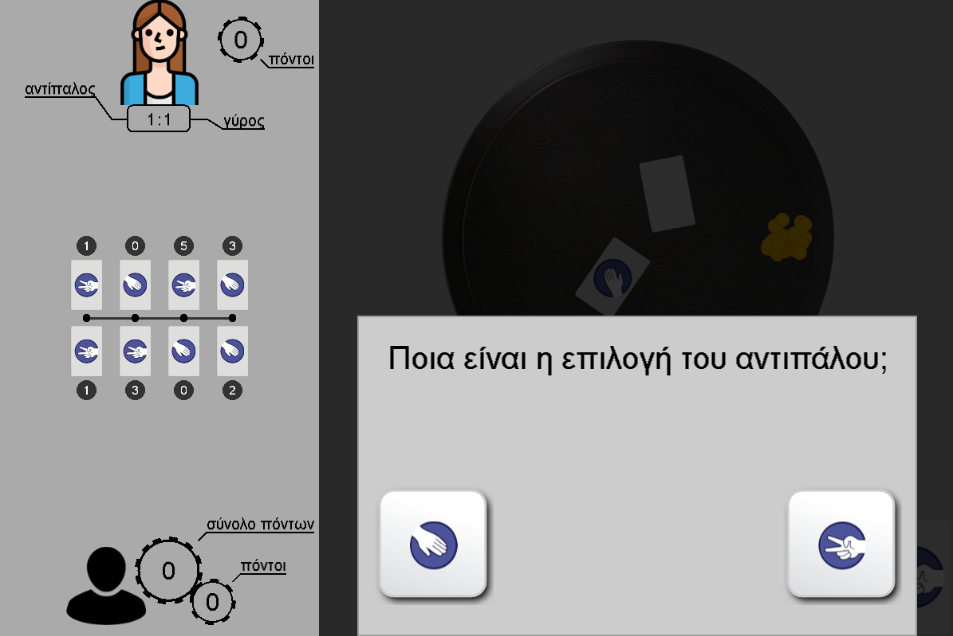
Participants are asked whether they can predict their opponents move or not (top figure) and then they are asked to register their prediction for the opponent’s move (bottom figure).
